# Simulation and analysis of spatio-temporal maps of gastrointestinal motility

**DOI:** 10.1186/1475-925X-7-2

**Published:** 2008-01-14

**Authors:** Wim JEP Lammers, Leo K Cheng

**Affiliations:** 1 Department of Physiology, Faculty of Medicine and Health Sciences, UAE University, Al Ain, United Arab Emirates; 2 Bioengineering Institute, The University of Auckland, Private Bag 92019, Auckland 1142, New Zealand

## Abstract

**Background:**

Spatio-temporal (ST) maps provide a method for visualizing a temporally evolving and spatially varying field, which can also be used in the analysis of gastrointestinal motility. However, it is not always clear what the underlying contractions are that are represented in ST maps and whether some types of contractions are poorly represented or possibly not at all.

**Methods:**

To analyze the translation from stationary or propagating rhythmic contractions of the intestine to ST maps, a simulation program was used to represent different patterns of intestinal contraction and to construct their corresponding ST maps. A number of different types of contractions were simulated and their ST maps analyzed.

**Results:**

Circular strong contractions were well represented in ST maps as well as their frequency and velocity. Longitudinal contractions were not detected at all. Combinations of circular and longitudinal contractions were, to a limited extent detectable at a point in space and time. The method also enabled the construction of specific ST-patterns to mimic real-life ST maps and the analysis of the corresponding contraction patterns.

**Conclusion:**

Spatio-temporal simulations provide a method to understand, teach and analyze ST maps. This approach could be useful to determine characteristics of contractions under a variety of circumstances.

## Background

Spatio-temporal (ST) maps provide a method for visualizing a temporally evolving and spatially varying field. In the gastrointestinal system, they were first used in 1997 TheyTheyto analyze intestinal motility [1]. To date, this type of analysis, also called D-maps (the D refers to the diameter), has been used to analyze the motility of the small intestine [2-4], colon [5], stomach [6], or of barium contents [7]. In most of these studies, recordings were obtained from experiments performed in vitro but recently in vivo recordings have also been obtained [8,9].

However, it is uncertain what a spatio-temporal map (ST map) may entail. For example, it is not certain what types of contraction are detected and how faithfully they can be represented by the ST maps or whether there is a bias for particular types of contractions. One way to start addressing these uncertainties is to use a simulation program, which allows systematic investigation of a number of different types of intestinal contractions, such as stationary or propagating segmental or pendular contractions, and to generate the corresponding ST maps. By comparing the original, albeit artificial, contractions with the derived ST map, it is possible to clarify some of the potentials and limitations of ST mapping.

## Methods

**Figure 1:**
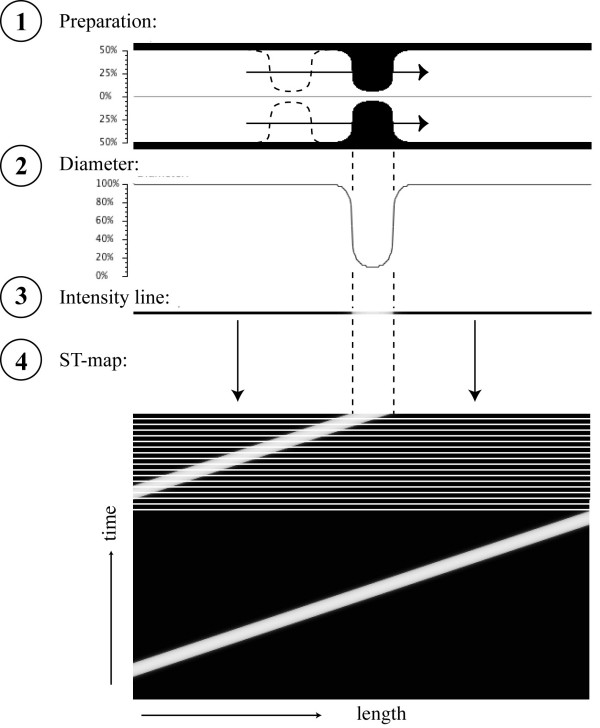
Simulation of contractions and creation of corresponding spatio-temporal (= ST) maps. In panel 1, a segment is depicted with a contraction propagating from left right. The intestinal diameter, as shown in panel 2, is 100% along the segment and decreases to 10% at the location of the contraction. Panel 3 shows an intensity line at this particular instance in time (white for total occlusion and dark for a 100% open lumen). At each time step, as the contraction propagates to the right, a new diameter curve is plotted, converted into an intensity line which is then stacked on top of the previous lines in the ST map (panel 4).

Simulations were performed using custom written software (SimST) [10]. The simulations are empirically based, temporally quasi-static and have a relatively limited physiological link to events occurring in the small intestine. They involved displaying a cross-section of an intestinal segment, inserting various types of stationary or propagating, stable or rhythmic constrictions, calculating the diameter of the segment, and plotting those values in an ST-map. (see Panels 1–5 in Figure 1). Variables which can be adjusted in the simulations to determine the resultant effects on the ST maps included the speed, the direction, the length, depth, and frequency of contractions, and the curvature or misalignment of the investigated sample.

In the first simulation (Figure 1), a single contraction occurred on both sides of the intestinal tube that constricted the lumen to 10% of its original value. The contraction propagated from left to right and, at this moment during the simulation, was located in the middle of the preparation. In panel 2, the diameter of the tube was calculated along its length and, for this time step, translated, as is the custom in ST mapping, in a single line whereby the value for the diameter was converted into the intensity of the line (panel 3). Black shading corresponded to an open tube (100%) whereas white shading represented a fully occluding contraction (0%).

Therefore, at this particular moment during the simulation, the "intensity line" was black along the length of the segment except for a middle portion that was white to light grey, indicating contraction in that area. This line was then stacked on top of previous lines obtained from previous time steps (panel 4). At the end of this simulation, in which two uniformly propagating contractions occurred after each other, two parallel white lines were obtained in the ST map. The angle of these lines indicates the direction and the velocity of the contraction while the vertical distance (time) between the lines is a measure of the frequency of this contraction.

In the ST maps presented in this article, time was oriented upwards in the vertical direction and space in the horizontal direction as is the convention of some groups [4,8]. However, it should be noted that there are alternative conventions to orientate time in the horizontal direction [1,3] or in the downward vertical direction [2,5].

## Results

A series of different controlled contraction types were systematically analyzed by varying a number of parameters that affected the motility patterns of intestine and examining the corresponding ST maps.

### Variations in velocities

**Figure 2:**
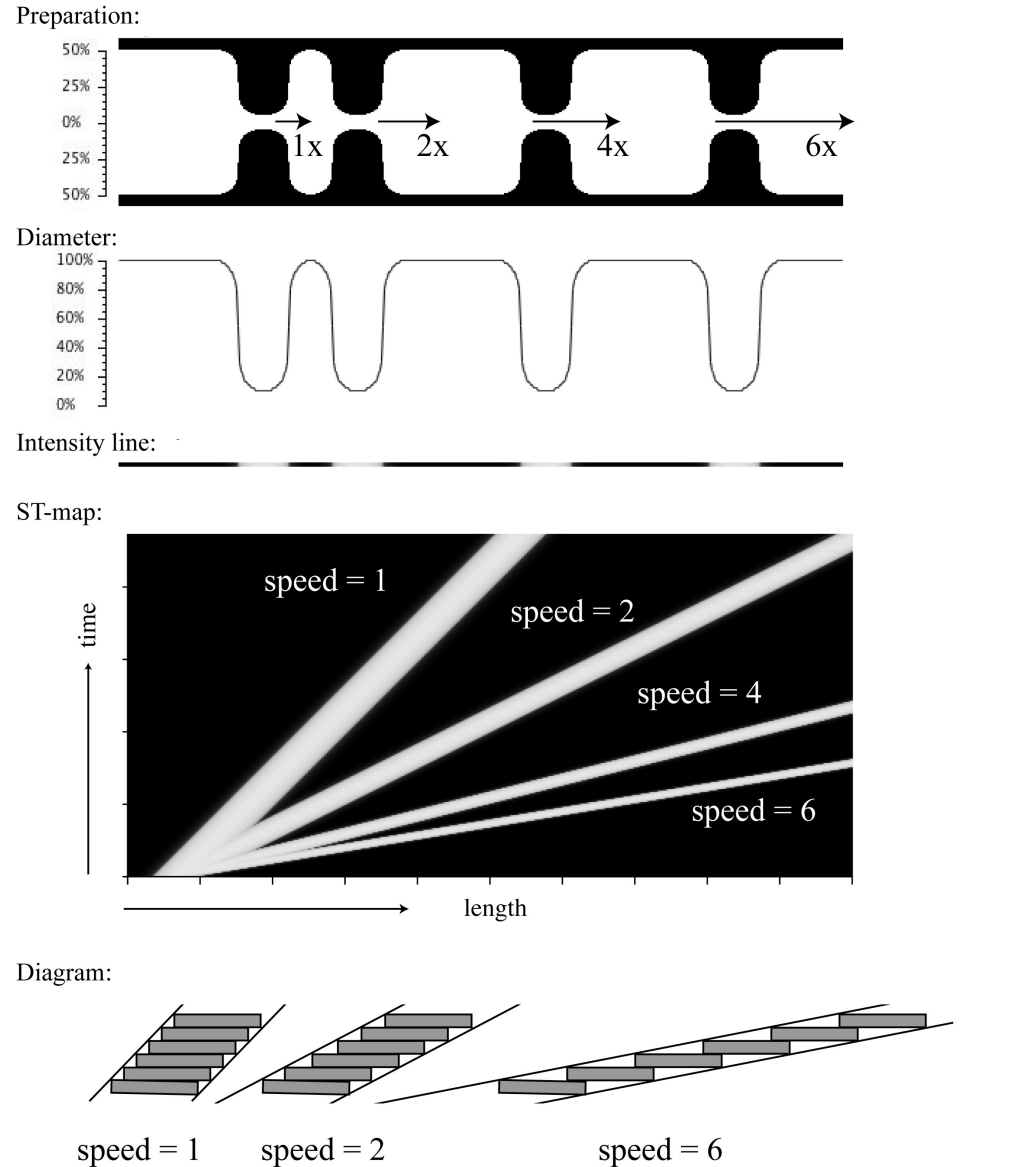
Effects of different velocities on ST map patterns. All contractions started simultaneously at the left but propagated at different velocities (1x, 2x, 4x and 6x). There is an appearance in the ST map that the slower contractions are more prominent than those created at faster speeds. This is an illusion as the shift between each time step is greater at higher speeds, resulting in a 'thinner' appearance (see lower schematic diagram).

In this first simulation, the control simulation introduced in the methods (Figure 1) was used again, but contraction velocities varied. Figure 2 shows the ST maps after this circular contraction had propagated at the following relative speeds: 1x, 2x, 4x and 6x. A cursory examination of the resultant ST map may suggest that the contraction at speed 6 is weaker than that at speed 1 due to the narrower white band. In actual fact, the contraction width and depth are exactly the same at all velocities. This appearance is caused by the larger spatial shift between time steps at higher velocities, which will make the overall line appear 'thinner' (refer to schematic diagram at the bottom of Figure 2). If the width of the contraction is measured strictly in the horizontal direction, the correct value is obtained. This physically means that, at higher velocities, the contraction spends less time at any location in the intestine.

### Stationary and propagating oscillating contractions

**Figure 3:**
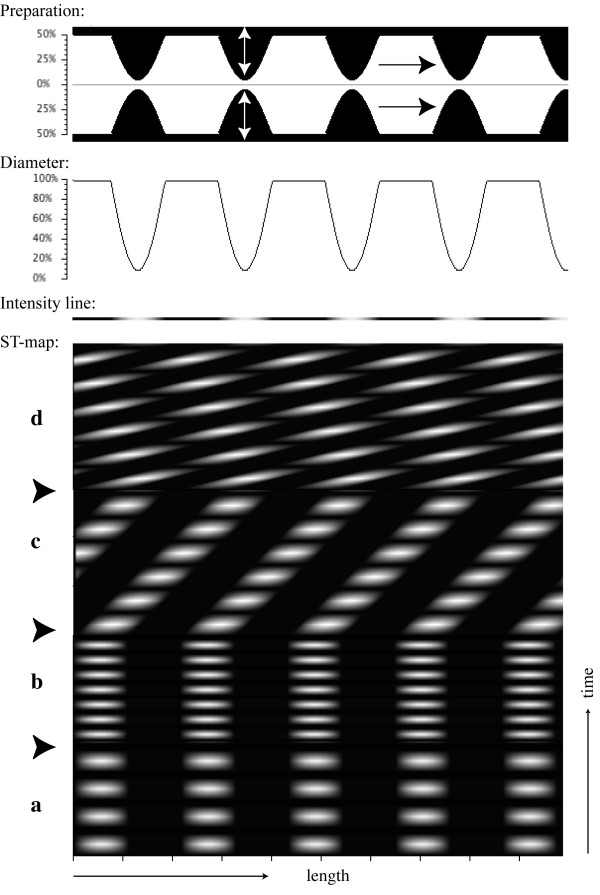
Variations in frequency and propagation of oscillatory contractions. In this simulation, contractions at five separate locations oscillated in the same rhythms up and down (white double arrow), mimicking contraction and relaxation (period a). In the next period (b) the frequency of oscillation was doubled. In period (c), the oscillations contracted at the first frequency while they also propagated to the right. In period (d) the speed of propagation was doubled.

In this simulation (Figure 3), contractions were allowed to contract and relax (i.e., oscillate) at five fixed locations. The contractions oscillated at two different frequencies (periods 'a' and 'b') and thereafter, the contractions were allowed to propagate towards the right at two different speeds (periods 'c' and 'd'). From the ST maps, in 'a' and 'b' the individual contractions, and in 'c', the direction of propagation can still be followed. This is more difficult to discern when the speed was doubled as shown in period 'd' (Figure 3). From the ST map, other directions of propagations could be imagined than the one that actually took place.

### Pendular contractions

In the small intestine, one of the most common types of contraction is the pendular contraction. This pendular contraction reflects the rhythmic contractions of longitudinal muscles that occur at the rhythm of the slow wave [11]. As analyzed and simulated by Melville et al. [12], this contraction, in itself, does not narrow the lumen. Therefore, as the inner diameter does not change, there are no resulting imprints in the ST maps, and this type of contraction occurring by itself remains invisible. Indirectly however, it could be possible to detect these pendular contractions if and when other circular contractions occur on top of the longitudinal contractions. This was analyzed in the following simulation.

**Figure 4:**
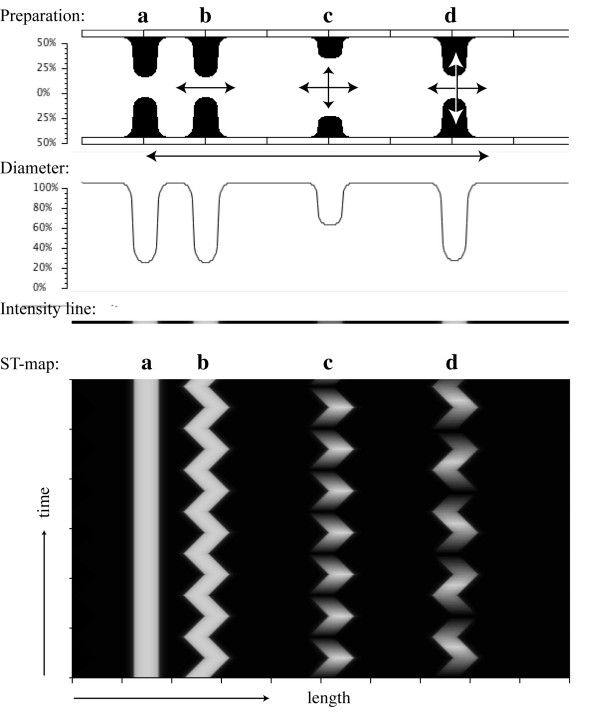
ST maps of pendular contractions. Pendular contractions occurring alone, as they do not narrow the lumen, cannot be detected in ST maps. However, if a circular contraction occurs on top of the pendular contractions, then pendular contraction can, to a degree, be reconstructed. Contraction 'a' was not fixed to the pendulating wall, remains static, and produced a straight line on the ST map. Contraction 'b', on the other hand, was fixed to the pendulating wall and therefore oscillated from right to left revealing the frequency and magnitude of the pendular contractions. In general however, circulating contractions are not stable but contract and relax, in 'c' in the rhythm of the pendular contraction and in 'd' at a different rate.

In the simulation (shown in Figure 4) the walls of the preparation oscillated left and right, thereby simulating the to-and-fro movements of pendular contractions. Several additional circular contractions were set to occur. These circular contractions fully occluded the lumen. In contraction 'a', the contraction was kept fixed, unrelated to the pendulating wall. This contraction therefore caused a straight line on the ST map. The second contraction 'b' was fixed to the pendulating wall and therefore oscillated left and right, thereby revealing in the ST map the frequency and the magnitude of the pendular swings. Contraction 'c' represented a more physiological case which included contraction and relaxation and, in this case, in the same rhythm as the pendular contraction. This created in the ST map a pattern of a shaded zigzag, whereby the horizontal excursions reflected the pendular swings, while the oscillations in intensity corresponded to the circular contractions. Finally, in contraction 'd', the circular contractions had a 1:3 rhythm to the pendular contractions.

### The effects of the curvature of the segment

**Figure 5:**
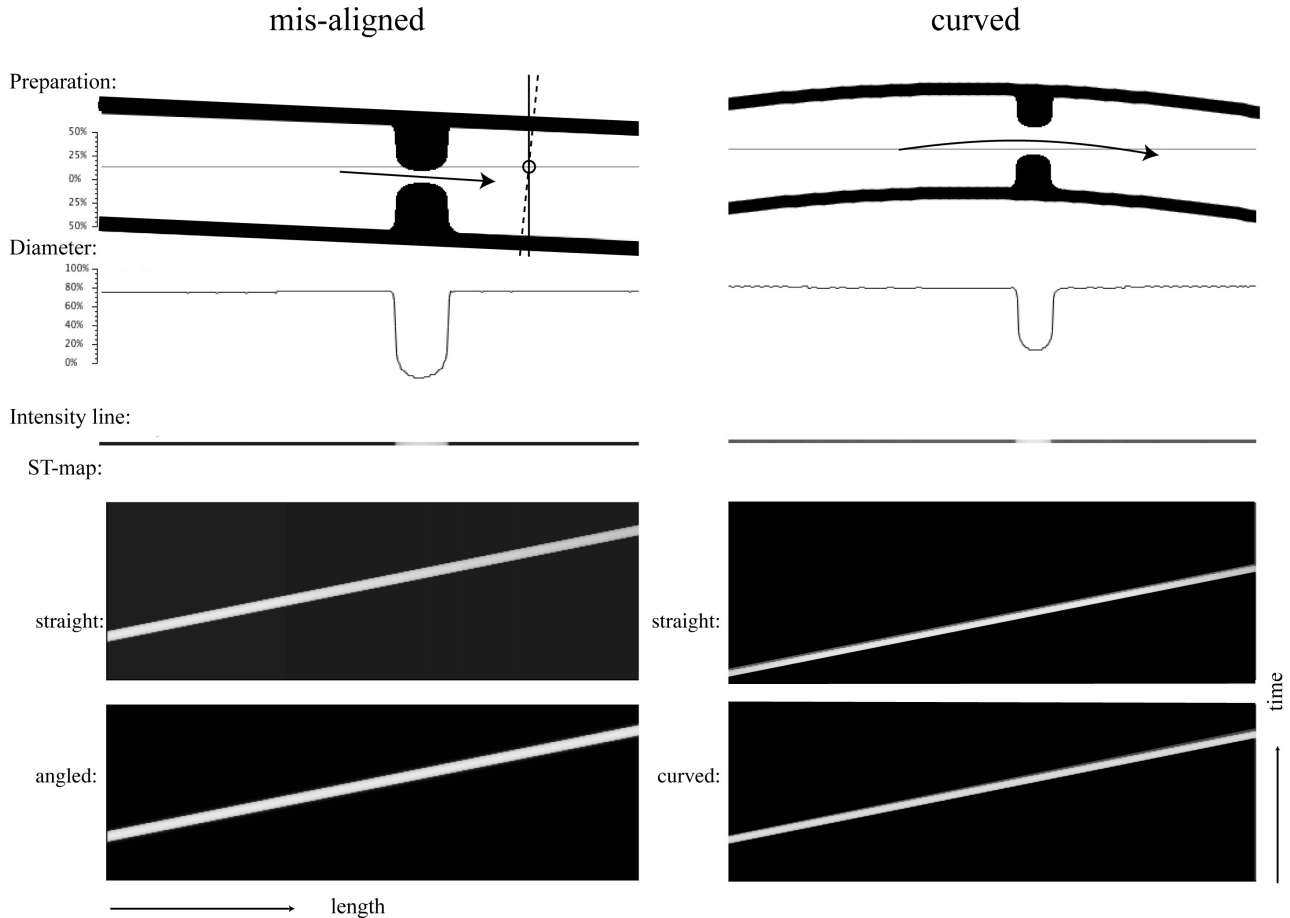
The influence of misaligned (left panels) and curved (right panels) intestinal segments on ST-profiles. The experiments were performed with straight and aligned segments (upper ST maps) and with the modified segments (lower ST maps). The width and slope of the propagating contractions in the ST maps were not greatly influenced by these modest misalignments or curvatures. The dashed and vertical lines in the misaligned preparation indicate the small error in estimating the diameter caused by this misalignment.

Often, with in vitro or in vivo experiments it is difficult or near impossible to perfectly align an intestine segment with a major axis. The intestinal segment being investigated will not be aligned perfectly straight but takes on an angle or a curvature, depending on the length and the amount of stretch exerted at both ends. The effect this misalignment of the intestine sample would have on ST maps was investigated in the following simulations. Two scenarios were studied as shown in Figure 5; a) a misalignment of a straight section and b) a curved segment. Each simulation was first performed with aligned and straight segments (upper ST maps) and then with the misaligned or curved segments (lower ST maps). As shown in the patterns of the imprints, the effect of curvature and misalignments are hardly visible in the ST maps. As indicated by the short vertical and dashed lines in the misaligned preparation, the lack of a strong effect is due to the small deviation in angle of the misaligned segment. In this simulation, the angle of misalignment was approximately 15%, resulting in an overestimated the diameter by about 2%, a difference that is not visible and well within experimental error. Similar results were obtained when the intestines were curved (Figure 5, right panels).

### Inverse Analysis

**Figure 6:**
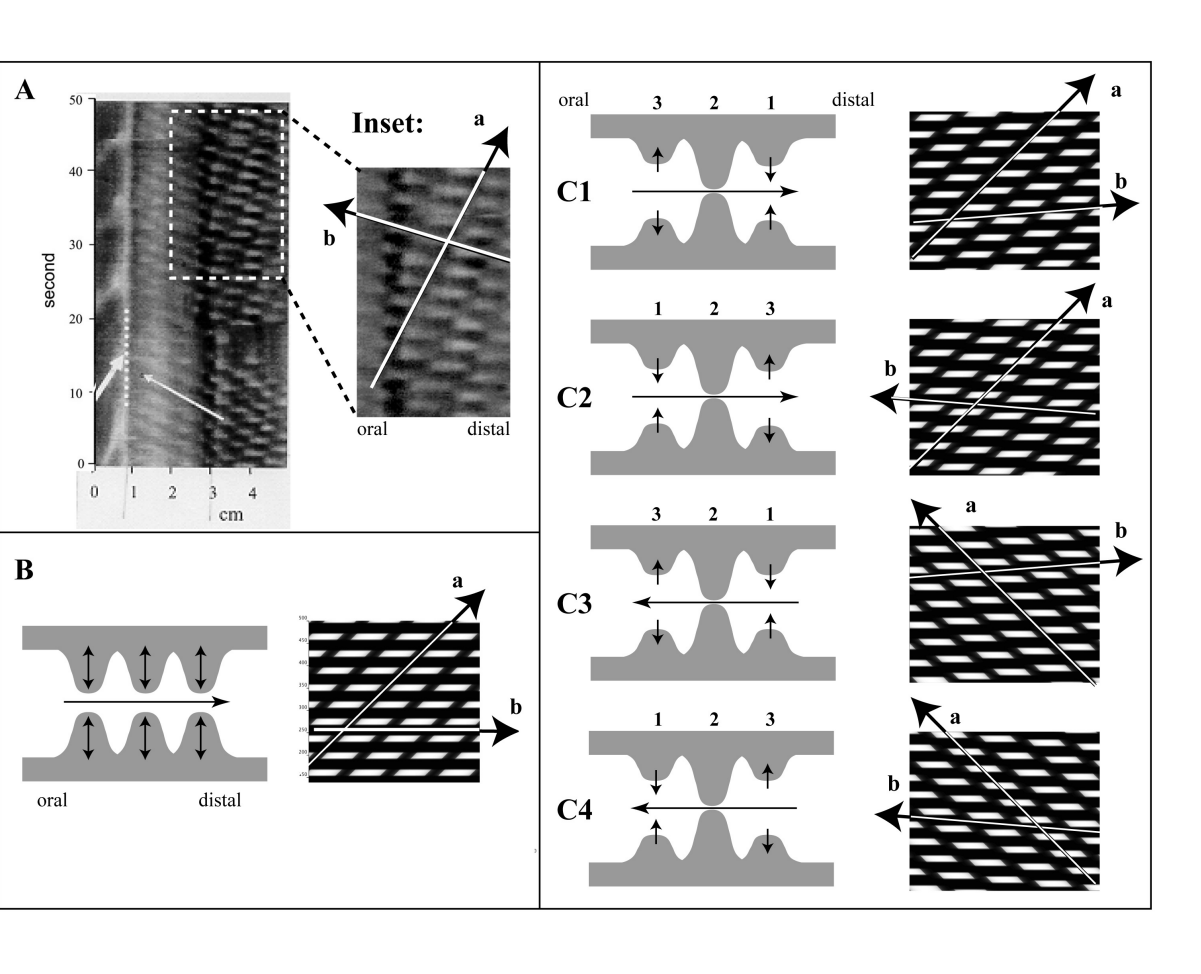
Inverse analysis of an experimentally recorded ST map. Panel A displays an ST map obtained fluoroscopically from a mouse in vivo (modified from [13] and used with permission from The American Physiological Society). A selected window (dashed square) is magnified at right (inset). The overall pattern is that of series of frequent and regular contractions in the duodenum. Two arrows indicate principle axes of the patterns; one with a steep slope ('a') and a second with a more gradual slope ('b'). Panel B displays a simple type of a simulated contraction pattern in which a burst oscillated at a width and a frequency similar to that in panel A. These oscillatory contractions also propagated in the aboral direction as indicated by arrow 'a' with a slope similar to that in the inset. Arrow 'b' however is horizontal as all oscillations occur at the same time, implying that the in vivo contractions did not occur simultaneously. In panels C, the contractions oscillated and propagated in various sequences. In C1, the contractions did not contract simultaneously, but one after the other, stepping forward in the aboral direction (indicated by '1', '2' and '3'). With this scenario, slope 'a' is similar but slope 'b' is directed in the aboral direction, opposite to that measured in vivo (inset). Scenario C2, in which the contractions occurred in the opposite sequence ('3', '2' and '1'), does produce a slope 'b' similar to the original one. Scenarios C3 and C4 repeat scenarios C1 and C2 but with propagation in the oral direction, creating a different slope of arrow 'a'.

The preceding series of analyses could be described as a type of 'forward' analysis in which a particular contraction is simulated to produce a corresponding ST map. The reverse, or 'inverse' scenario can also be performed. This was done using an experimental recording from a mice duodenum (Figure 6 panel A) in which the contractions of the duodenum were measured in vivo by fluoroscopy [13]. The inset shows a magnified view of this ST map displaying a regular pattern of contractions. The aim of this simulation was to generate an ST map that would mimic as close as possible this real life ST map. From the original recording, it is clear that the contractions were frequent and regular, and there were two principal components to ST maps as shown by the arrows in panel A. The axis shown by arrow 'a' provides an indication of the speed of the global movement of the waves, while the axis indicated by arrow 'b' indicates the degree of regularity or delay between neighboring contractions.

The ST maps were initially simulated with a regular burst of occluding contractions (panel B). The size of the imprints was best resembled by setting the width of the contractions at 70% of the distance between neighboring contractions. This produced an ST map (panel B) in which the width and the frequency of the individual contractions resembled those in the original record. The contractions also propagated in the aboral direction as indicated by arrow 'a'. The propagation speed was adjusted to produce a slope similar to slope 'a' in the inset. In addition, a second arrow 'b' was drawn connecting the neighboring contractions. The slope of this arrow was horizontal as all contractions occurred simultaneously. As the slope of arrow 'b' in the inset was obviously not horizontal, it was concluded that neighboring contractions did not occur simultaneously. Several scenarios were tested as shown in panel C. In these simulations, the contractions again propagated while oscillating at the same frequency. The timing of neighboring contractions was then offset with respect to each other. In panel C1, the contraction started at '1', was complete at '2' and relaxed at '3'. The slope of arrow 'b' was therefore towards the aboral direction. This was however opposite to that measured in the inset. If the contraction sequence was reversed (scenario C2) then the correct slope was obtained. In the original paper that described this recording [13], it was assumed that propagation actually was retrograde (white arrow in the duodenum in panel A). This was tested in scenarios C3 and C4 in which the propagation was in the oral direction and the contraction sequence took place in either the oral or in the aboral direction. From this analysis, it would now seem that the contraction propagation was actually in the aboral direction while the sequence of contractions occurred in the oral direction.

**Figure 7:**
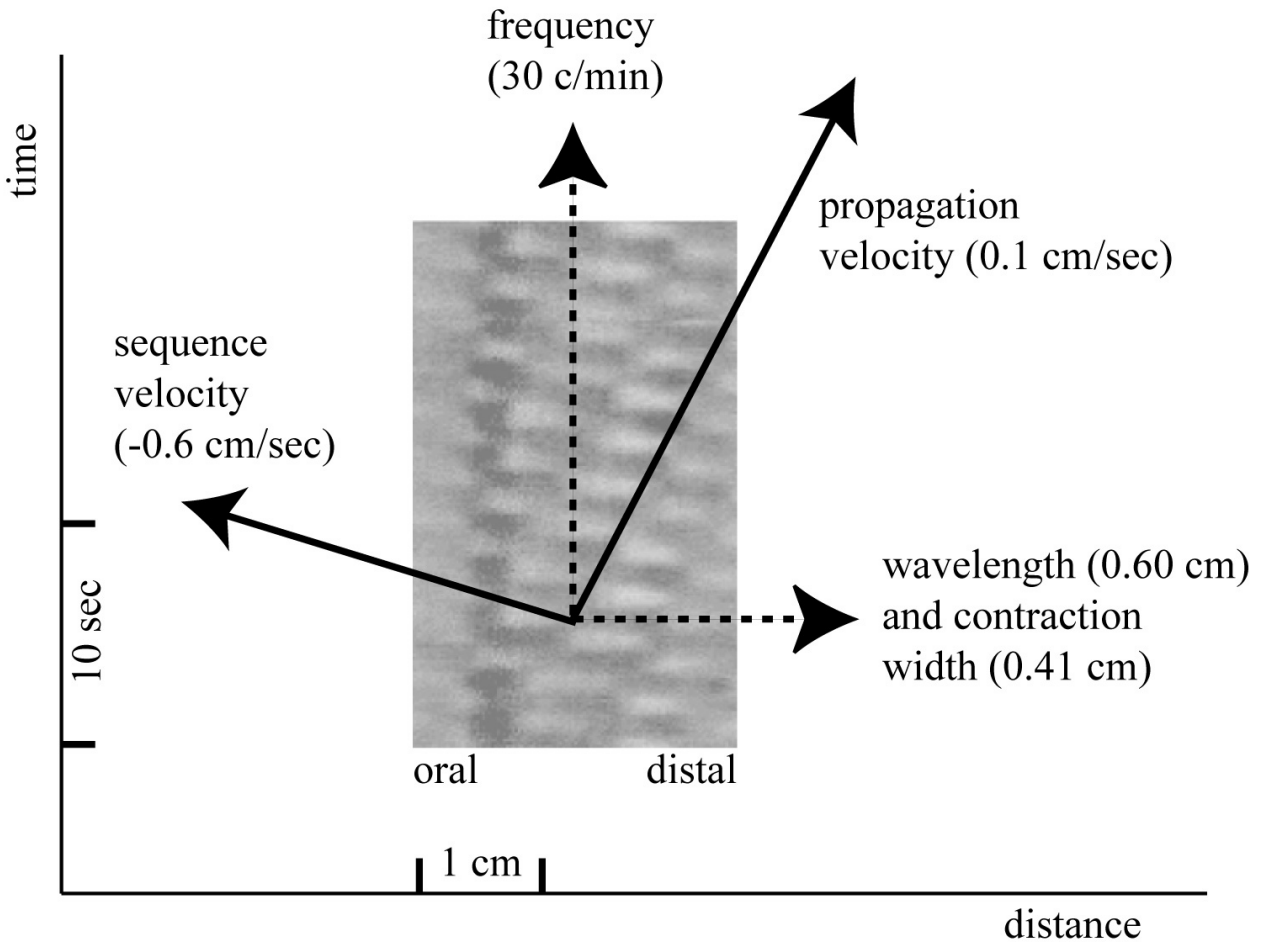
Following the analysis of figure 6, several parameters of contraction can now be derived from the ST-map such as the frequency of contraction, the distance between one contraction and the next (= wavelength), the width of the contraction, the aboral propagation velocity and the sequence velocity. The sequence velocity is negative as the contractions step backwards in the oral direction.

Following this analysis, it is now possible to calculate actual parameters from the ST-maps. As shown in the diagram in Figure 7, the length of the contraction and the distance between one contraction and the next can be calculated together with the frequency of the contractions. The two slopes "a" and "b" can be used to calculate the velocity of propagation and the velocity of the sequence of contraction, which is negative in this case as the sequence progressed in the oral direction.

## Discussion

This analysis has shown some of the potentials and limitations of spatial-temporal mapping of intestinal contractions. ST mapping is at its best in determining origin and direction of fully occluding circular contractions. In several cases, the frequency, velocity and width of strong circular contractions can be measured accurately. On the other hand, contractions of the longitudinal muscle layer, as in pendular contractions, are not detected at all (Figure 4). Combinations of circumferential and longitudinal contractions can partly be reconstructed depending on their corresponding propagations and frequencies (Figure 3). Finally, this approach also presents the potential of inverse analysis of real-life ST maps and the characterization of the underlying contraction patterns (Figure 6).

In many cases, video recordings of the intestinal tube are used to measure the diameter of the segment and in most cases; it is the outer diameter, not the inner luminal diameter, that is used for the construction of ST maps. In smaller animals with thin luminal walls, the difference between the two might be negligible, but, in larger animals and in humans, this difference may become significant. Another approach has been to use the variations in contrast in computer-enhanced images as a way to obtain signals suitable for ST analysis [4]. However, the relation between contrast, contraction and diameter has not been evaluated. For example 'contrast' recordings may also be able to pick up longitudinal contractions that diameter recordings may miss. Finally, especially in *in vivo *fluoroscopy studies, it is often not the intestinal wall but the intestinal contents that are measured [7,13]. It is known that changes in luminal content do not necessarily reflect muscular contractions [14] but the effects a local contraction may have on luminal contents located further away have not been analyzed.

Nevertheless, within these constraints, ST mapping is a useful tool to detect and characterize intestinal contractions, provided that its limitations are known. Together with other techniques, such as intra-luminal pressure recordings, impedance measurements of intestinal flow and myo-electrical recordings, it contributes to our increasing knowledge of normal and abnormal intestinal behavior.

## Conclusion

Simulations of intestinal contractions and the creation of corresponding spatio-temporal maps (ST maps) have shown that some types of contractions, especially circular contractions, are well represented in ST maps while others, especially contractions of the longitudinal muscle coat, are not detected by ST maps at all. Combinations of circular and longitudinal contractions can, to a certain extent, be detected. This approach also enables the construction of specific ST-patterns to mimic real-life ST maps and the analysis of the underlying contraction patterns.

## Competing interests

The author(s) declare that they have no competing interests.

## Authors' contributions

WJEPL created the software and performed the simulations presented in this study. LKC and WJEPL wrote the manuscript. Both authors read and approved the final version of the manuscript.
